# Palliative Care in Africa and the Caribbean

**DOI:** 10.1371/journal.pmed.0010005

**Published:** 2004-10-19

**Authors:** Dingle Spence, Anne Merriman, Agnes Binagwaho

## Abstract

In many of the world's poorest countries, dying is often accompanied by avoidable pain and other distressing symptoms. How can we improve care at the end of life?

## Teaser

It must be made a public health priority

*“If someone is condemned to a premature death because of the injustice of global health inequality, it is doubly unjust for that person to be condemned to an agonising death racked by preventable pain.”* [1]

In many resource-poor countries, death is accompanied by avoidable pain and other distressing symptoms. Unfortunately, governments in these countries usually give care at the end of life a low priority compared with preventive and curative services [2]. This prioritization makes little sense, especially when applied to treating patients with cancer and HIV/AIDS, since prevention efforts are often failing to reduce the disease burden, while treatments aimed at cure or prolonging life are still too expensive to be made widely available.

As three physicians in Jamaica, Uganda, and Rwanda, we believe that providing quality care at the end of life should be seen as a global public health priority. By using relatively low-cost palliative care approaches and community-based strategies, thousands of terminally ill patients in Africa and the Caribbean could be relieved of their pain and suffering.

## The Burden of Cancer and HIV/AIDS

In the countries where we work, the burden of cancer and HIV/AIDS is overwhelming. In Africa about 2.5 million people die annually from HIV/AIDS, and more than 0.5 million die from cancer [3,4]. Sepulveda and colleagues have estimated that each year, at least one in 200 people in the five African countries that they studied (Botswana, Ethiopia, Tanzania, Uganda, and Zimbabwe) need palliative care at the terminal stages of HIV/AIDS or cancer [2]. This figure does not include those needing palliative care for other diseases or those suffering from a serious illness in the pre-terminal stages. Thus, perhaps one in 100 people in these countries needs some level of palliative care each year [2].

In Rwanda, as in most other African countries, infectious diseases are still rife. Health professionals are often faced with the terrible dilemma of having to choose between saving lives and easing the suffering of the dying. Indeed the authorities usually believe that any investment in palliative care would be at the expense of providing life-saving treatments for those suffering from curable, often infectious illness.

In many Caribbean countries, while the scourge of water- and insect-borne infectious diseases is largely under control, the prevalence rates of HIV in the adult population are some of the highest in the world [5]. In Jamaica, the largest English-speaking country in the Caribbean (population 2.5 million), in 2001, there were an estimated 20,000 people living with HIV and 980 deaths from AIDS [6]. Further, Jamaica's proximity to the United States means that many people aspire to a lifestyle more representative of a wealthy, industrialized nation, and are thus susceptible to diseases such as cancer, coronary artery disease, and diabetes. Unfortunately, the island's struggling public health system is often unable to provide adequately for patients with these diseases.[Fig pmed-0010005-g001]

**Figure pmed-0010005-g001:**
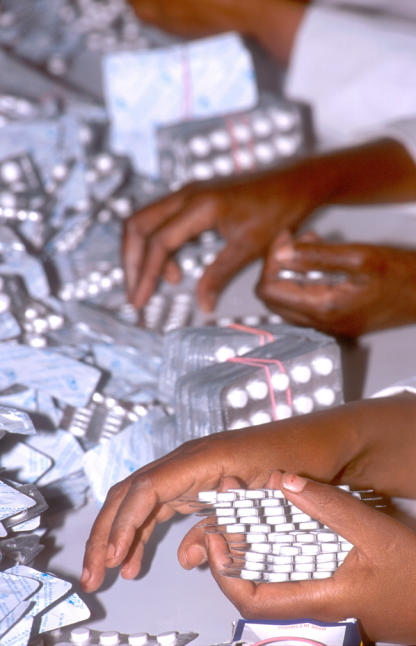
Better distribution of analgesics would improve palliative care provision

## The Arguments for Palliative Care

Prevention efforts—including health promotion, education, and screening—and treatments aimed at cure or prolonging life are key strategies needed to reduce the burden of HIV/AIDS and cancer in resource-poor countries [7]. However, when it comes to prevention, in many countries the effects of health education, health promotion, and screening programs have yet to make an impact on rates of HIV infection or cancer. When it comes to treatment, the provision of high-quality, affordable treatments for patients with HIV/AIDS and cancer requires the development of appropriate and accessible infrastructure and technology with sustainable funding.

At present, access to treatment where we are working is essentially controlled by the ability of the patient to pay. Thus, only about one in 200 people with HIV in Uganda are able to obtain antiretroviral medicines [8]. Furthermore, patients in developing countries often present with far advanced malignant disease, and as many as 80% of people with cancer may be incurable at diagnosis [9].

Given that prevention isn't taking effect in many places, and curative services are poorly available or inappropriate, we believe that the provision of palliative care (Box 1) in the Caribbean and Africa should be viewed as an urgent public health problem. About 80% of cancer patients will have pain in the terminal phase of their disease [1], and we estimate that at least 25% of HIV/AIDS patients have substantial pain during their illness.

**Box 1. The WHO Definition of Palliative Care**

The WHO has defined palliative care as an approach that improves the quality of life for patients and their families facing the problems associated with life-threatening illness, through the prevention and relief of suffering. This is done through early identification, careful assessment, and treatment of pain and other problems—physical, psychological, and spiritual. Dying is regarded as a normal process, and death is neither hastened nor postponed [2]. The philosophy of hospice and palliative care acknowledges death, dying, and bereavement as a reality of life.

Effective and relatively cheap methods exist for controlling pain and other symptoms. For example, the World Health Organization (WHO) has outlined a relatively cheap way of relieving cancer pain in about 90% of patients, which could be extended to patients with HIV/AIDS [2]. Sadly, most people in Africa and the Caribbean who need pain relief aren't receiving it [10].

## Assessing Patients' Needs

Several studies in East Africa have looked at the experience of dying, the quality of care at the end of life, and patients' unmet needs [2,11,12]. Recurring themes are (1) unmet physical needs, including the need for relief of pain and other symptoms, (2) the need for food, (3) the high cost or unavailability of appropriate analgesic drugs, (4) the severe financial constraints on the family and caregivers, (5) the need for training of family caregivers, (6) lack of psychosocial support, and (7) social isolation due to the stigma attached to a diagnosis of HIV/AIDS.

In the Caribbean, patients' needs at the end of life appear to be similar to those of patients in many East African countries. A qualitative study in Grenada, in the Eastern Caribbean, showed that people preferred to die at home rather than in hospital and—in the absence of pain relief and much-needed counseling, information, and financial support—they took solace in spiritual comfort [13]. In Jamaica (Box 2), although data are scarce, it seems that patients' needs are very similar to those in Grenada. Christianity is the principal religion of Jamaica, and faith in God and family support are critical factors in patient care at the end of life. Outside of the hospital setting, appropriate analgesics are difficult to access and are often unaffordable. Patients and caregivers are not provided with enough information to help them understand disease processes, and what to expect as the ill person nears death. There is little or no palliative care provision for patients with HIV/AIDS.

**Box 2. Dying in Jamaica—A Typical Case Scenario**

*This fictional case scenario gives an impression of the sorts of problems that patients face at the Hope Institute, Kingston—Jamaica's first public hospice.*

A 50-year-old woman is diagnosed with inoperable lung cancer. Because of brachial plexus involvement, she experiences severe pain and weakness of her arm. She is treated at Kingston Public Hospital with palliative radiotherapy, which helps the pain for a few months. But then the pain returns, and she requires a high dose of slow-release morphine for pain control.

She lives in the mountains, and her house is a two-and-a-half-hour bus ride from Kingston, the capital city. Unfortunately, the public pharmacy in Kingston is unwilling to dispense more than a week's supply of morphine at any one time, because they have limited supplies (there is a shortage of the drug in Jamaica) and because they think the patient's dose is unacceptably high. So she has to make the exhausting five-hour round trip every week.

Her husband's health has also recently declined, and the woman's sister now has to care for the patient and her husband. The family now has the financial means to afford only one small meal a day, and they rely on donations from their church community in order to survive.

Because the family's savings dwindle, and the public pharmacy faces further shortages of morphine, the woman with cancer requires multiple admissions to the hospice in Kingston over the last six months of her life in order to get suitable analgesia.

## Uganda's Public Health Approach

Uganda has made palliative care for patients with AIDS and cancer a priority in its National Health Plan [10]. In 1993, after conducting a feasibility study, Hospice Uganda was established in Kampala, making palliative care available to a population of about 2 million people (Uganda's population is 22 million people). There are now two other hospices, one in Mbarara serving 1 million people, and one in Hoima serving 350,000 [8]. The hospice care provided by these units is all home-based care. This type of care provision is designed to meet the cultural and practical needs of the people in Uganda, where most people prefer to die in their own homes, and where people are often buried in their household gardens.

Hospice Uganda provides community-based care principally to patients suffering from HIV/AIDS and cancer. Almost all patients coming to the hospice have pain, and a great deal of attention is focused on good pain management. Uganda is only the third African country to have made morphine available and affordable to its patient population. Because of the dearth of legal prescribers (doctors, dentists, and vets only), in May 2004, Uganda changed the statute. This allowed midwives to prescribe pethidine, and allowed clinical palliative care nurses and clinical officers who are specially trained and registered to prescribe morphine.

**Figure 1:**
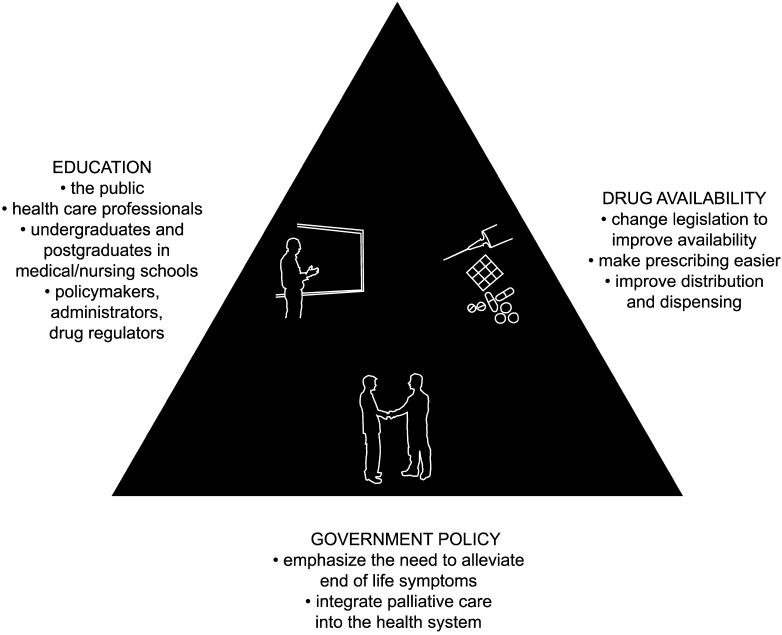
The WHO's Triangle of Foundation Measures (Adapted with permission from “A Clinical Guide on Supportive and Palliative Care for People with HIV/AIDS” [http://hab.hrsa.gov/tools/palliative/])

How was Uganda—an African country with a relatively under-funded health service—able to provide a palliative care service? A national program using a public health approach to reach those in need was established following principles outlined in the WHO's National Cancer Control Guidelines [4]. These guidelines outline the importance of assessing the magnitude of the problem, setting measurable objectives, evaluating possible strategies, and choosing priorities for initial activities. A series of workshops were held in Uganda between 1998 and 2000, where the WHO's “little cost, big effect” measures began to be addressed. The three key measures involve education, increased drug availability, and changes in government policy (Figure 1).

## Other African Initiatives

Four other African countries—Botswana, Ethiopia, Tanzania, and Zimbabwe—have made the development of home-based care a priority in dealing with the HIV/AIDS epidemic [2]. Botswana has an operational home-based care program integrated into its national health system, while in the other three countries, care is largely provided through private organizations. But few of the home-based care services in these countries include the capacity for providing effective pain relief [2].

## The Next Steps

By using strategies such as providing access to an essential short list of relatively cheap generic medications, and other methods recommended by WHO, it has now been proven that palliative care in the African context is affordable and achievable [2,7,14].

We believe that, following the Ugandan and Botswanan models, palliative care should be integrated into national government strategies. In order to begin to show governments the importance and economic justification for developing a palliative care health policy, it is clear that needs assessments are an essential first step. It is likely to be much less expensive to provide community-based care with family and community support at the end of life than to burden already overcrowded hospital wards with patients suffering end-stage disease. There is a long tradition, both in Africa and in the Caribbean, of caring for the disabled, the mentally ill, and the young and elderly sick at home.

Both start-up and sustainable funding are enormous issues that will need to be addressed by local governments, international funding agencies, and charitable bodies. Advocating palliative care to decision makers, providing training programs for health professionals, and making medications available and affordable are important challenges.

Research in individual countries is needed to assess whether the above recommendations are suitable locally. Hospice Africa Uganda is advocating to other African governments and assessing other African countries where local laws and customs may dictate the most suitable way to provide palliative care together with government support. Partnerships and a public health approach to palliative care must be the way forward.

**Palliative Care Resources for the Developing World**

**African Palliative Care Association**

Representing Kenya, South Africa, Tanzania, Uganda and Zimbabwe E-mail: apca@hospiceafrica.or.ug

**Hospice Africa (Uganda)**

Resource and Training Centre PO Box 7757, Kampala, Uganda Tel: +256 41 266 867 / 510089; Fax: +256 41 510087—residence E-mail: info@hospiceafrica.or.ug; E-mail: anne@hospiceafrica.or.ug

**Centre for Palliative Learning**

Hospice Association of the Witwatersrand PO Box 87600, Houghton, Johannesburg 2041, South Africa

**Hospice Information**

At http://www.hospiceinformation.info. Click on “Training” to search for courses and conferences in palliative care and bereavement. Requires member's password to access this part of the website but membership is free to people in developing countries—contact hospice information at + 44 (0)870 903 3 903 (telephone), + 44 (0)20 8776 9345 (fax), or info@hospiceinformation. Information is also circulated quarterly by E-mail to members under the title of e-Choices.

**Palliative Care in Resource-Poor Settings**

A freely available overview of HIV/AIDS palliative care, written by Kathleen Foley, Felicity Aulino, and Jan Stjernswärd. At http://hab.hrsa.gov/tools/palliative/chap19.html.

**Living Well with HIV/AIDS**

A freely available manual on nutritional care and support for people with HIV/AIDS, by the Food and Agriculture Organization of the United Nations. At http://www.fao.org/DOCREP/005/Y4168E/Y4168E00.HTM.

**Cancer Pain Relief: A Guide to Opioid Availability**

A section of this guide, by the WHO, is freely available at http://www.medsch.wisc.edu/painpolicy/publicat/cprguid.htm.

## Abbreviation

WHO: World Health Organization

## References

[pmed-0010005-b1] Singer PA, Bowman KW (2002). Quality of care at the end of life. BMJ.

[pmed-0010005-b2] Sepulveda C, Habiyambere V, Amandua J, Borok M, Kikule E (2003). Quality care at the end of life in Africa. BMJ.

[pmed-0010005-b3] World Health Organization (2001). World health report 2001. Mental health: New understanding, new hope.

[pmed-0010005-b4] World Health Organization (2002). National cancer control programmes: Policies and managerial guidelines, 2nd ed.

[pmed-0010005-b5] Joint United Nations Programme on HIV/AIDS and World Health Organization (2002). AIDS epidemic update: December 2002. http://www.who.int/hiv/pub/epidemiology/epi2002/en/.

[pmed-0010005-b6] Avert.org (2004). Caribbean statistics summary. http://www.avert.org/caribbean.htm.

[pmed-0010005-b7] World Health Organization (2003). Project description: A community health approach to palliative care for HIV and cancer patients in Africa. http://www.who.int/cancer/palliative/projectproposal/en/.

[pmed-0010005-b8] Merriman A, Heller KS (2002). Hospice Uganda—A model palliative care initiative in Africa. An interview with Anne Merriman. Innov End-of-Life Care.

[pmed-0010005-b9] World Health Organization (1996). Cancer pain relief with a guide to opioid availability, 2nd ed.

[pmed-0010005-b10] Stjernsward J (2002). Uganda: Initiating a government public health approach to pain relief and palliative care. J Pain Symptom Manage.

[pmed-0010005-b11] Murray SA, Grant E, Grant A, Kendall M (2003). Dying from cancer in developed and developing countries: Lessons from two qualitative interview studies of patients and their carers. BMJ.

[pmed-0010005-b12] Kikule E (2003). A good death in Uganda: Survey of needs for palliative care for terminally ill people in urban areas. BMJ.

[pmed-0010005-b13] Kreitzschitz S, Cox Macpherson C (2003). End of life care. Perspectives from families and caregivers. West Indian Med J.

[pmed-0010005-b14] Merriman A (2002). Palliative medicine: Management of pain and symptoms for cancer and/or AIDS patients in Uganda and other African Countries, 3rd edition.

